# Process mining and customer journey mapping in healthcare: Enhancing patient-centred care in stroke rehabilitation

**DOI:** 10.1177/20552076241249264

**Published:** 2024-05-16

**Authors:** Ingy Shafei, Jonathan Karnon, Maria Crotty

**Affiliations:** 1 1066 The University of Adelaide, Adelaide, SA, Australia; 2 1065 Flinders University, Adelaide, SA, Australia

**Keywords:** Cardiovascular disease, stroke, artificial intelligence, digital health, health, technology, rehabilitation, lifestyle, outcomes, quantitative, connected care, personalised medicine

## Abstract

**Background:**

Patient-centred care and enhancing patient experience is a priority across Australia. Stroke rehabilitation has multiple consumer touchpoints that would benefit from a better understanding of customer journeys, subsequently impacting better patient-centred care, and contributing to process improvements and better patient outcomes. Customer journey mapping through process mining extracts process data from event logs in existing information systems discovering patient journeys, which can be utilized to monitor guideline compliance and uncover nonconformance.

**Methodology:**

Utilizing process mining and variant analysis, customer journey maps were developed for 130 stroke rehabilitation patients from referral to discharge. In total, 168 cases from the Australasian Rehabilitation Outcomes Centre dataset were matched with 6291 cases from inpatient stroke data. Variants were explored for age, gender, outcome measures, length of stay and functional independence measure (FIM) change.

**Results:**

The study illustrated the process, process variants and patient journey map in stroke rehabilitation. Process characteristics of stroke rehabilitation patients were extracted and represented utilizing process mining and results highlighted process variation, attributes, touchpoints and timestamps across stroke rehabilitation patient journeys categorized by patient demographics and outcome variables. Patients demonstrated a mean and median duration of 49.5 days and 44 days, respectively, across the patient journeys. Nine variants were discovered, with 78.46% (*n* = 102) of patients following the expected sequence of activities in their stroke rehabilitation patient journey. Relationships involving age, gender, length of stay and FIM change along the patient journeys were evident, with four cases experiencing stroke rehabilitation journeys of more than 100 days, warranting further investigation.

**Conclusion:**

Process mining can be utilized to visualize and analyse patient journeys and identify gaps in service quality, thus contributing to better patient-centred care and improved patient outcomes and experiences in stroke rehabilitation.

## Introduction

The quadruple aim of health, aiming to optimize health systems performance, is looking at enhancing the patient experience, population health, reducing costs and healthcare provider's work towards optimizing healthcare delivery, patient outcomes and quality of care.^1,2^

Patient experiences are developed through their encounters and interactions across the care continuum provided by the healthcare provider and mapping this contributes to visually externalizing this patient experience to the provider to improve the quality of care.^3^ This can provide healthcare providers with knowledge and data on patient interactions and touchpoints through the different stages of the customer life cycle.^4,5^ A customer journey (CJ) is defined as ‘a collection of interactions (or touchpoints) between a customer and a firm’.^6^ A tool for understanding the CJ is CJ mapping (CJM), which visually maps the steps and each interactive touchpoint as customers go through the journey when engaging with a service provider and assists service providers in understanding the customer experience at the different points of interactions in their journey with the provider.^5,6^

There are several different types of journey mapping diagrams including CJ, Experience Map, Mental Model Diagram, Service Blueprint and spatial maps utilizing diverse sources of data such as observations, questionnaires, and interviews.^5^ However, these types of manual processes involved in journey mapping are tedious and often subjective.^4^

Process science refers to ‘the broader discipline that combines knowledge from information technology and knowledge from management sciences to improve and run operational processes’^7^^(p.811)^. Process mining is a bridge between process science and data science that enables mapping CJ maps by extracting knowledge about process execution from the data (event logs) that is already available in existing information systems. This includes data generated and stored in organizational databases for all activities that have been implemented in a process, such as enterprise resource planning systems, customer relationship management and systems and hospital information systems.^4^

Several studies have applied process mining for CJM in the area of healthcare.^4,8–11^ To date, process mining has not been utilized to map CJs in stroke rehabilitation settings. The current research aims to address this gap by utilizing process mining for stroke rehabilitation patients in South Australia, aiming to contribute to enhancing patient-centred care and improving the quality of the stroke rehabilitation processes.

## Background

Firstly, it is important to consider the background for stroke rehabilitation, the concept of CJM with respect to patient experience and quality improvement and process mining with its various techniques in healthcare.

### Stroke rehabilitation

Stroke is a major contributor to death and disability worldwide. In 2019, the global prevalence of stroke globally was 101.5 million people.^12^ In 2018, an estimated 387,000 people had a stroke at some time in their lives in Australia. In 2017, there were around 38,000 stroke events, more than 100 every day.^13^ In 2019, stroke was the second leading cause of disability-adjusted life-years worldwide in both the 50–74 years and 75 years and older age groups.^14^ Early and intensive rehabilitation has contributed to the recovery of motor function capacity in patients.^15^

### CJM and quality improvement

Improving the customer's experience is one of the leading priorities across many industries^4^ and healthcare providers across Australia want all patients to have the best possible hospital experiences.^16^ Mapping patient journeys contributes to identifying process variations and improving patient outcomes and healthcare service quality. Patient journeys in a healthcare setting include many activities, both administrative, for example, registrations, admissions, and discharge among others as well as clinical activities, for example, diagnostic tests and scans, triaging, and surgeries.^17^ Within process mining research, several elements of CJM have been contextualized.^4,6,18,19^ In the study conducted by Bernard and Andritsos,^18^ CJM components were identified to include the customer, journey, mapping, goals, touchpoints, timeline, channel, stage, experience, lens and multimedia (detailed in Table 1).

**Table 1.** Components of customer journey mapping (CJM).^4,18^

Customer	The customer is ‘the stakeholder experiencing a service’.
Mapping	Mapping is a ‘process consisting of tracking and describing customers’ responses and experiences when using a service’.
Goal	A CJ should be mapped with a goal in mind (main intention) as this stimulates user actions and thought processes.
Touchpoint	A touchpoint is ‘an interaction between customers and companies’ products or services’. The sequence of touchpoints can be iterative, where customers can go to the same touchpoint several times. They can also be nonlinear, where customers can miss a touchpoint or even quit early.
Timeline	A timeline describes ‘the duration of the journey from the first until the last touchpoint’. Timestamps are often present and a touchpoint sequence can be established.
Channel	A channel is ‘the method chosen by the customer to interact with the touchpoint’ such as through social media or websites or call centres.
Stage	A stage encompasses several touchpoints and these can be before, during, and after the experience. In some instances of CJM, stages are not used at all.
Experience	Includes a customer's feedback as well as emotions involved in the journey.
Lens	Layers through which multiple views can occur.
Multimedia	Multimedia usage in the CJ can include audio, video, photos, etc.

CJMs perceived by providers often differ from ones mapped through evidence collected by leveraging customer traces and data in information systems.^18,19^ Establishing the goal in CJM provides the organization and the customer the opportunity to set a common target for the analysis of the CJ.^4^ Consumer journeys utilizing tools such as process mining, can support multiple ways to discover, monitor, and improve processes based on these real event logs. Utilizing process mining aids in discovering processes by providing a visual ‘as-is’ representation of the journey.^18,19^

Within the healthcare context, outcomes are of paramount importance thus identifying bottlenecks, resource analysis, process deviations, conformance checking and working towards reducing process complexities among many other applications for process mining in CJM, has a direct outcome on enhancing the CJs in clinical settings.^18,19^

### Process mining

Process science and business process management are used to explore organizational processes. Data science looks at producing value from data by generating knowledge from datasets. Process mining is a bridge between these two disciplines, aiming to discover, monitor and improve processes by extracting knowledge about process execution from the data (event logs) that is already available in existing information systems.^4^ Van der Aalst first introduced process mining in 1999 in the Netherlands, where event logs are used to extract knowledge that allows process discovery, conformance, and enhancement.^20,21^ In the last decade, process mining has become widely used both in industry and academia empowering organizations to streamline and improve business processes.^22^

There has been an evident increase in the use of process mining in healthcare settings due to the complexity of the healthcare process. Process mining can utilize big data available in hospitals to identify and provide statistics and models of patient journeys.^17,23^ In addition, it can be utilized to monitor compliance with guidelines, standards, policies and procedures^4^ as well as uncover nonconformance and provide predictions and recommendations.^24^

Three principal types of process mining have been exposed in the literature: process discovery, conformance checking, and enhancement.^20^ Process models are extracted from event logs using process discovery; process conformance checking uncovers process deviations against predefined process models and guidelines and process enhancement further expands existing models utilizing additional information in event logs.^20^ In addition, there are four analysis perspectives that can be utilized in process mining, including control flow, organizational, case and time perspectives. Control flow concentrates on the order of the activities, organization concentrates on related resource information, case concentrates on case attributes and time focuses on event timing and frequency journeys.^17,23^

In terms of process mining applications and research, while many studies have been conducted in healthcare,^25^ and four studies in stroke,^26–29^ process mining has not been applied to stroke rehabilitation.

## Methodology

The goal of the research within the current context was to discover and analyse the stroke rehabilitation patient CJ aiming to improve patient outcomes, service quality and patient experiences. This case study utilized process discovery and variant analysis for the patient journey for stroke rehabilitation starting from the date of referral to the end date of rehabilitation, and explored demographic variants, for age and gender groups, as well as variants in outcome measures, for length of stay (LOS) and functional independence measure (FIM) change.

### Research questions

For this purpose, clinical processes for stroke rehabilitation patients are analysed and the research seeks to answer the following questions:

Q1: What process characteristics of stroke rehabilitation can be extracted and represented from the Australasian Rehabilitation Outcomes Centre (AROC) data utilizing process mining?
Q2: What variation can process mining reveal across stroke rehabilitation patient journeys categorized by patient characteristics (age, gender, and baseline FIM), LOS and FIM outcomes?

### Nature of the study

The study is exploratory in nature, with the goal of identifying the processes undertaken by stroke rehabilitation patients and variations in outcomes and demographics across their journeys. The case study was conducted between 2017 and 2019, and the sites involved included Flinders Medical Centre, the Repatriation General Hospital, the Royal Adelaide Hospital and Hamstead Hospital in South Australia, Australia. Variables of interest for the study were identified and the data was extracted from the AROC dataset and merged with inpatient data for patients admitted for stroke. The AROC^30^ is a national benchmarking system that collects outcome data for inpatient and ambulatory rehabilitation settings aiming to improve clinical outcomes and provide information on intervention efficacy (South Australia Department of Health State-wide Service Strategy Division 2009). The project was reviewed and approved by the SA Department for Health and Ageing Human Research Ethics Committee. The study utilized de-identified historical data collected across the hospitals, and full ethics approval was granted including a waiver of consent and meeting requirements of the ‘National Statement of Ethical Conduct in Human Research’ in Australia.

### Context of the study

Within the current research context, the customer is the Stroke Rehabilitation patient. The patient journey was defined by tracing the Stroke Rehabilitation patient events from the referral date to the end date. An event log was defined, which included all events performed in this timeframe.

The mapping within the current research context was implemented utilizing process mining to create process maps. Disco^31^ one of the prominent commercial tools, was used due to its ease of use, where it is often utilized for initial analyses in process mining.^22^

Touchpoints in CJM are a set of interactions (events) where the patient might be involved during the healthcare journey. Timeline refers to ‘the end-to-end timestamps registered in order to track when a distinct touchpoint occurs and its order’^4^^(p.4)^. Within the current context, all event interactions with available timestamps in this healthcare journey for stroke rehabilitation patients from the referral date to the end date were included. Finally, the ‘channel’ is the method by which the customer has interacted with the touchpoints and within the context of this research, the AROC and inpatient stroke datasets will be used to extract relevant timestamps and patient attributes. Stage in CJM involves grouping touchpoints into clusters of activities, thus including subprocesses.

### Data and event log construction

The current case study for stroke rehabilitation patients is utilizing data that included clinical episodes from the AROC dataset from January 2017 to December 2019. This dataset included 168 cases, with 1848 events executed and 11 main process activities. Additional patient activities/attributes were included from the inpatient stroke data from January 2017 to December 2019 which included 6291 cases.

#### Creation of event log

An event log was created utilizing the event ID (Master Key), activity/attributes (attributes included summarised in Table 2) and time stamps for activities (time stamps utilized are summarized in Table 3).

**Table 2.** Activity/attributes included in process mining.

Length of stay (LOS)	This is a derived data item calculated as the episode end date minus the episode begin date, minus the number of leave days during the episode’ (AROC^30^^(p.111)^).
FIM change (FIMchangeto)	Functional independence measure (FIM) change is the difference between the patient's discharge FIM scores and their admission FIM scores (AROC^30^^(p.110)^).
Hospital	Hospital is defined at the moment of the episode.
Ward on admission	Patient ward on admission is defined at the moment of the episode.
Ward on discharge	Patient ward on discharge is defined at the moment of the episode.
DRG	Patient's diagnosis-related group
Principal diagnosis	Patient's principal diagnosis at the moment of the episode.
Age	Age is defined at the moment of the episode.
Gender	The biological differences between males and females, as represented by a code (AROC^30^^(p.15)^).

**Table 3.** Time stamps for activity.

Onset date (OnsetDate):	This date defines ‘the date of the injury or impairment that has directly driven the need for the current episode of rehabilitation’ (AROC^30^^(p.41)^).
Acute admission date (AcuteAdmDate):	This date defines ‘the date of the acute admission relevant to the current episode of rehabilitation’ (AROC^30^^(p.43)^).
Referral date (Referral):	This date defines ‘the date that the rehabilitation team received a referral for the patient’ (AROC^30^^(p.24)^).
Assessment date (Assessment):	This date defines ‘the date the patient was first seen by a clinician or the rehabilitation team to assess their appropriateness for rehabilitation care’ (AROC^30^^(p.25)^).
Clinically rehab ready date (Clinically Rehab Ready Date):	This date defines ‘when the rehabilitation physician, or physician with an interest in rehabilitation, deems the patient ready to start their rehabilitation program and have documented this in the patient's medical record’ (AROC^30^^(p.26)^).
Episode begin date (BegDate):	This date defines ‘the beginning of the rehabilitation episode and is the date from which length of stay (LOS) calculation begins’ (AROC^30^^(p.33)^).
Team planning date (TeamPlanDate):	This date defines ‘the date the multidisciplinary team rehabilitation plan was first recorded’ (AROC^30^^(p.40)^).
Date of starting FIM (StartFIMdate):	This date defines ‘the date that the patient's admission Functional Independence Measure scores were completed’ (AROC^30^^(p.47)^).
Date of ending FIM (EndFIMdate):	This date defines ‘the date the patient's discharge FIM scores were scored’ (AROC^30^^(p.51)^).
Date of clinically discharge ready (ClinicallyDischargeReady):	This date defines the date where ‘a patient should be defined as ready to be discharged to the community (Community Ready*)’ (AROC^30^^(p.53)^).
End date (EndDate):	This date defines ‘the date that the patient completes their rehabilitation episode. This date defines the end of the rehabilitation episode and is the date at which the length of stay (LOS) concludes’.

The patient is community-ready when he/she ‘no longer requires the intensity of therapy provided by an inpatient rehab service’ or ‘has achieved a level of function that allows them to be safely discharged to the community based on their dwelling/social/geographical/financial status’ or ‘the level of function is stable enough to enable prediction of long term support needs (if required)’ or ‘is medically stable (including comorbidities) and can be managed in the community by a GP’.

#### Preparing data for process mining tool

**Figure 1.**
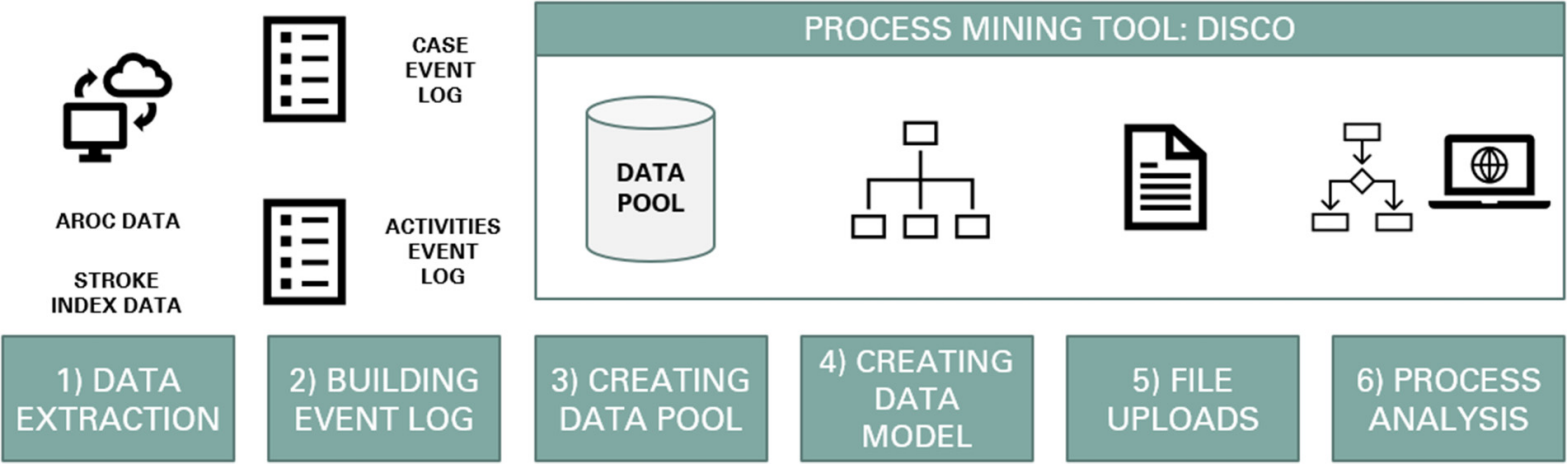
Steps in data processing and process analysis.

To upload the data into the process mining tool Disco, the process involved several steps (detailed in Figure 1); including data extraction, building the event log, data pool creation, data model creation, file uploads and process analysis.

Data extraction involved extracting data from AROC data and inpatient data for patients admitted for stroke from the time periods of 2017–2019. Data for time stamps and relevant activity/attributes was extracted to two Excel files; File 1: Master key + attributes (from inpatient stroke data) and File 2: Master key + relevant time stamps and attributes (from AROC data).

**Figure 2.**
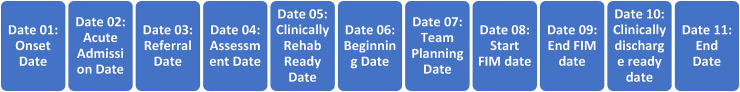
Stroke rehabilitation patient journey from onset to discharge.

The current data model detailed in Figure 2 was designed and confirmed by rehabilitation clinicians. The model for the patient journey in stroke rehabilitation commences with the onset of stroke injury driving the need for the rehabilitation episode. Subsequently, patients are admitted (with acute admission date recorded. Once admitted, a triage assessment is within 48 h of admission, and rehabilitation options, expected LOS and patient requirements are discussed. All stroke patients are to receive a rehabilitation plan unless they are end-of-life patients or a patient declines rehabilitation. Patients are deemed ready to start rehabilitation (with the clinically rehab-ready date recorded) and subsequently, the rehabilitation episode commences. The episode's begin date marks the beginning of the rehabilitation episode and LOS is calculated from that day. Once the patient undergoes rehabilitation, FIM starts and end dates are noted too. For patient discharge, a patient should be ready to be discharged to the community, that is, community ready, the date is noted inpatient episode and subsequently a patient completes the rehabilitation episode, and this (end-date) is the date at which the LOS concludes on completion patient is discharged.^30,32^

With often multiple events occurring on the same date, events were ordered as per the current data model detailed in Figure 2 for uploading. The datasets included 168 cases from the AROC dataset and were matched and merged with 6291 cases from inpatient data for patients admitted for stroke. The data was uploaded into a data pool in STATA and merged using the MasterKey. After merging the two datasets, 130 stroke rehabilitation patient episodes were used to develop CJ maps from referral to discharge. The data was configured in STATA into the format required by the process mining software tool Disco. Event log attribute categories were created for FIM change categories (0 = −50 to −1 change in FIM; 1 = 0–49 change in FIM; 2 = 50–100 change in FIM)), LOS (1 = 1 to 49 LOS in days, 2 = 50 to 99 LOS in days, 3 = 100 to 150 LOS in days) and age (1 = 1 to 49 years old, 2 = 50 to 100 years old). An example of a case log at the case identifier level can be seen in Table 4. Subsequently, the data was uploaded onto Disco.

**Table 4.** Example of case log at case identifier level.

Master key	Timestamp	Date	LOS	FIM change	Hospital	DRG	Ward on admission	Ward on discharge	Principal diagnosis	Age	Sex	FIM change category	LOS category	Age category
15003431	01	19/9/17	27	30	18	B70A	RA	RA	I639	81	2	1	1	2
15003431	02	19/9/17	27	30	18	B70A	RA	RA	I639	81	2	1	1	2
15003431	03	20/9/17	27	30	18	B70A	RA	RA	I639	81	2	1	1	2
15003431	04	21/9/17	27	30	18	B70A	RA	RA	I639	81	2	1	1	2
15003431	05	21/9/17	27	30	18	B70A	RA	RA	I639	81	2	1	1	2
15003431	06	21/9/17	27	30	18	B70A	RA	RA	I639	81	2	1	1	2
15003431	07	22/9/17	27	30	18	B70A	RA	RA	I639	81	2	1	1	2
15003431	08	22/9/17	27	30	18	B70A	RA	RA	I639	81	2	1	1	2
15003431	09	18/10/17	27	30	18	B70A	RA	RA	I639	81	2	1	1	2
15003431	10	18/10/17	27	30	18	B70A	RA	RA	I639	81	2	1	1	2
15003431	11	18/10/17	27	30	18	B70A	RA	RA	I639	81	2	1	1	2

LOS: length of stay; DRG: diagnosis-related group; FIM: functional independence measure.

## Statistical analysis

The current research evaluated the proposed research questions through the application of process mining to discover patients’ journey maps utilizing a log of 130 patient episodes undergoing stroke rehabilitation and results are demonstrated below through descriptive statistical analysis. Overall, 130 patients demonstrated a mean and median duration of 49.5 days and 44 days, respectively. Several patient characteristics and outcome variables were utilized in the current study, including age, gender, LOS and FIM change. Of the 130 patients, there were 53.85% males and 46.15% females. Age featured two categories of cases, with a frequency of 6.92% and 93.08% of cases in categories 1 and 2, respectively (1 = 1 to 49 years old, 2 = 50 to 100 years old). FIM change featured three categories of cases, with a frequency of 3.08%, 90.77% and 6.15% of cases in categories 0, 1 and 2, respectively (0 = −50 to −1 change in FIM; 1 = 0–49 change in FIM; 2 = 50–100 change in FIM). LOS featured three categories of cases, with a frequency of 81.54%, 17.69% and 0.77% of cases in categories 1, 2 and 3, respectively (1 = 1 to 49 LOS in days, 2 = 50 to 99 LOS in days, 3 = 100 to 150 LOS in days).

## Results

The current research utilized process discovery and variant analysis to understand how process mining detects and extracts process characteristics for stroke rehabilitation patients. In addition, control flow, time and case perspectives were utilized to address the research gaps and answer the research questions in the current study. Clinical pathways are complex and ultimately flexible, given patients present different needs, co-morbidities, and complications. Process variant analysis contributes to uncovering variations and differences in patient pathways to further investigate and analyse areas where process improvements can occur.^33^ In the current study, process variant exploration looks at variations between discovered variants executing similar clinical pathways.

Q1: What process characteristics of stroke rehabilitation can be extracted and represented from the AROC data utilizing process mining?

**Figure 3.**
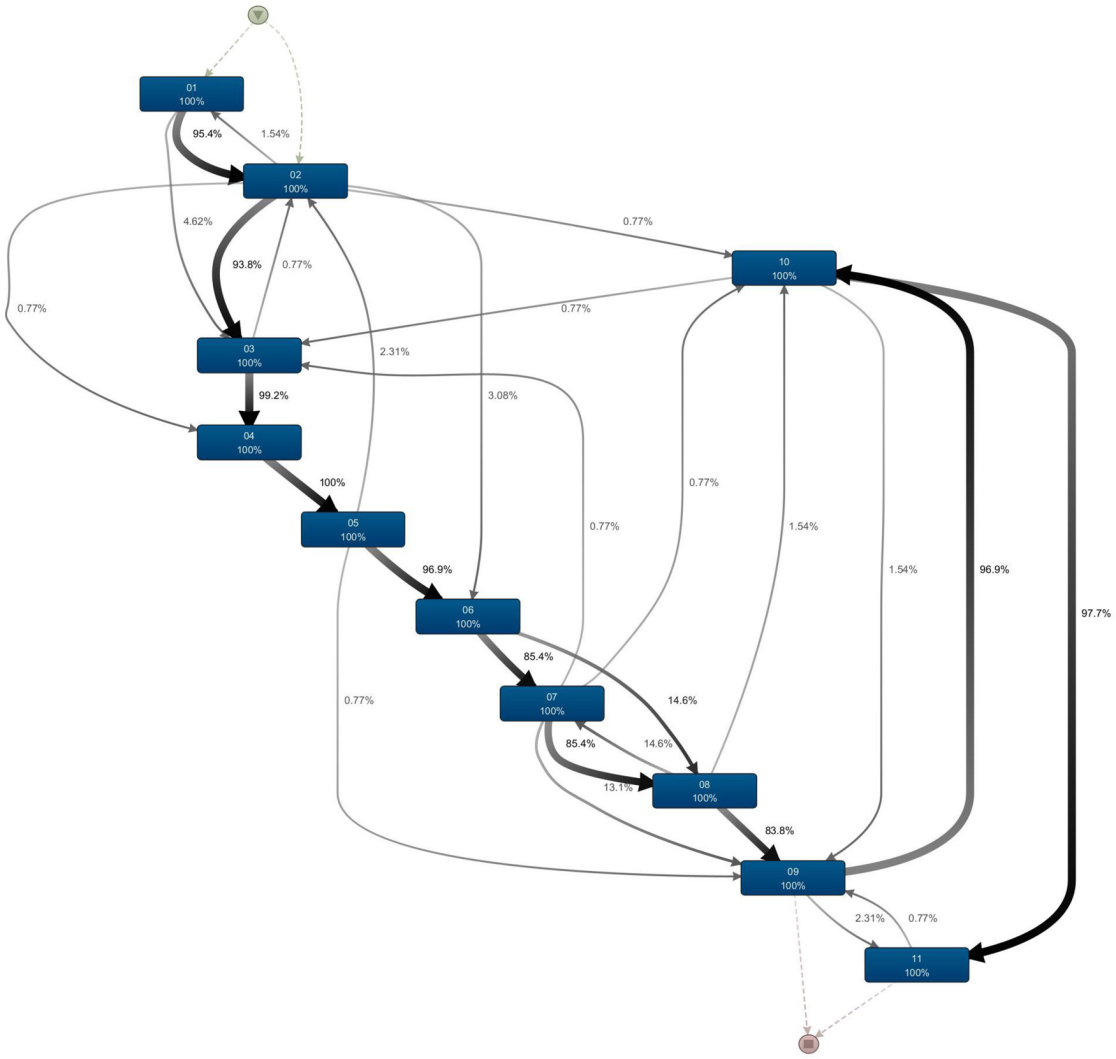
Disco-generated spaghetti diagram.

*Note*. The figure demonstrates the discovered process model. This complex and unstructured process seen in the figure appears as a ‘spaghetti process’, with the simplified steps seen in Figure 2.

**Figure 4.**
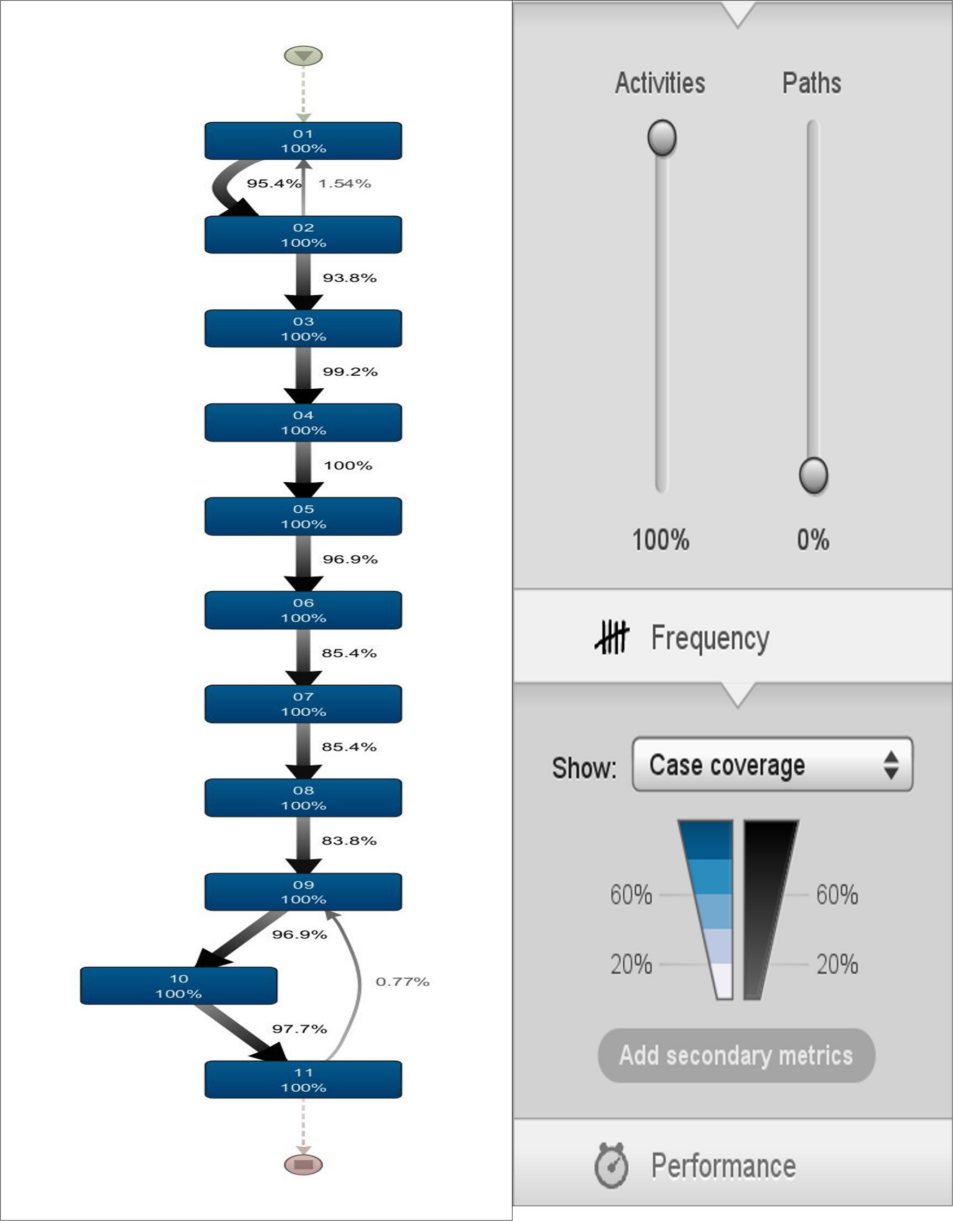
Disco-generated map for stroke rehabilitation customer journey.

*Note*. Disco tool functionalities displayed in the left part of the figure can be moved between 0% and 100% to discover and display the 11 stages of a stroke rehabilitation patient. Each of the rectangles in the figure represents one of the stages in the stroke rehabilitation journey and the distinct 11 stages of the stroke rehabilitation patient can be seen.

The process model for stroke rehabilitation was extracted and demonstrated the different touchpoints in the stroke rehabilitation CJ. The stroke rehabilitation clinical pathway was mapped and analysed through process mining analysis. The model is detailed in Figure 2 including the onset date, acute admission date, referral date, assessment date, clinically rehabilitation ready date, team planning date, beginning date, start and end FIM dates, clinically discharge ready date and finally end date. Process mining analysis was subsequently used to discover and analyse each patient's rehabilitation journey. The process model in Figure 2 provides a simplified view of a stroke patient's rehabilitation journey utilizing process mining. There is often a continuum of processes for patient journeys, and often complex and unstructured processes are seen (spaghetti processes).^34^ With the complexity of processes across hospital sites, the discovered process model appears spaghetti-like^23^ and the spaghetti process can be seen in Figure 3. The model was obtained using the heuristic miner with default settings. Note that some low frequent behaviour has already been filtered out, that is, the real process is even more Spaghetti-like than the model shown in Figure 4. This illustrates the challenges related to process mining when applied to less structured processes.

**Figure 5.**
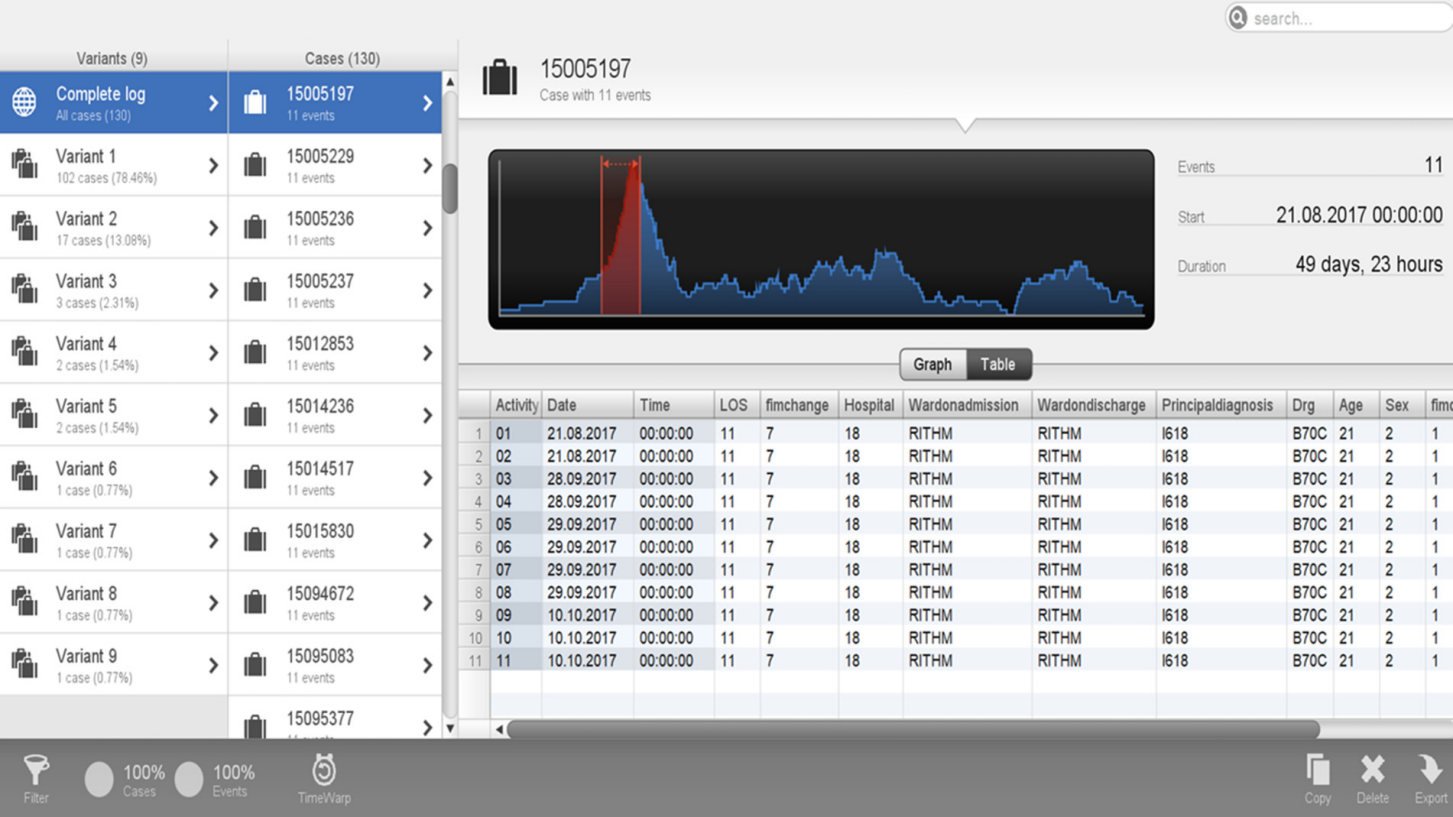
Complete case log for variant analysis in stroke rehabilitation (Variants 1–9).

*Note*. The different variants (Variants 1 to 9) in the study can be seen in the left column of the figure. Activities 01 to 11 can be seen in the table in the right bottom columns of the figure. The frequency of the nine variants in the left column of the figure was 78.46%, 13.08%, 2.31%, 1.54%, 1.54%, 0.77%, 0.77%, 0.77%, and 0.77%, for Variants 01 through 09, respectively. Variant 1 comprised 102 cases (78.46%). All patients in Variant 1 followed the expected sequence of activities in their patient journeys, as follows, Activity 01 is the onset date, Activity 02 is the acute admission date, Activity 03 is the referral date, Activity 04 is the assessment date, Activity 05 is the clinically rehab ready date, Activity 06 is the episode begin date, Activity 07 is the team planning date, Activity 08 is the date of starting FIM, Activity 09 is the date of ending FIM, Activity 10 is the date of clinically discharge ready, Activity 11 is the end date. Variants demonstrating different orders of events can be seen in Table 5.

Utilizing process variant exploration, nine variants were discovered in the current case study, illustrated in Figure 5. This demonstrates the case view in the Disco tool, which drills down into individual case levels. This enables detailed analysis of the individual cases, history, attributes, and deviations from the described process that might warrant further action in the clinical setting. The column labelled ‘variants’ in Figure 5 demonstrates the different variants’ specific sequences of activities and are sorted by their frequency. The cases that follow the same sequence of activities are subsequently placed and demonstrated in the list of cases in the second column named ‘cases’.

**Table 5.** Variant analysis in stroke rehabilitation (variants 1–9): sequence of activities Date 01 to Date 11.

Discovered sequence of activities	Variant 1 sequence of activities	Variant 2 sequence of activities	Variant 3 sequence of activities	Variant 4 sequence of activities	Variant 5 sequence of activities	Variant 6 sequence of activities	Variant 7 sequence of activities	Variant 8 sequence of activities	Variant 9 sequence of activities
Date 01	Date 01	Date 01	Date 01	Date 01	Date 02	Date 01	Date 01	Date 01	Date 01
Date 02	Date 02	Date 02	Date 03	Date 02	Date 01	Date 02	Date 03	Date 02	Date 02
Date 03	Date 03	Date 03	Date 04	Date 03	Date 03	Date 10	Date 02	Date 03	Date 06
Date 04	Date 04	Date 04	Date 05	Date 04	Date 04	Date 03	Date 04	Date 04	Date 08
Date 05	Date 05	Date 05	Date 02	Date 05	Date 05	Date 04	Date 05	Date 05	Date 07
Date 06	Date 06	Date 06	Date 06	Date 06	Date 06	Date 05	Date 06	Date 06	Date 03
Date 07	Date 07	Date 08	Date 07	Date 07	Date 07	Date 06	Date 07	Date 07	Date 04
Date 08	Date 08	Date 07	Date 08	Date 08	Date 08	Date 07	Date 08	Date 08	Date 05
Date 09	Date 09	Date 09	Date 09	Date 10	Date 09	Date 08	Date 09	Date 10	Date 09
Date 10	Date 10	Date 10	Date 10	Date 09	Date 10	Date 09	Date 10	Date 11	Date 10
Date 11	Date 11	Date 11	Date 11	Date 11	Date 11	Date 11	Date 11	Date 09	Date 11

The different variants (1 to 9) in the study can be seen in the horizontal axis of the table. Variant 1 comprised of 102 cases (78.46%). All patients in variant 1 followed the expected sequence of activities in their patient journeys, as follows, Activity 01 is the onset date, Activity 02 is the acute admission date, Activity 03 is the referral date, Activity 04 is the assessment date, Activity 05 is the clinically rehab ready date, Activity 06 is the episode begin date, Activity 07 is the team planning date, Activity 08 is the date of starting FIM, Activity 09 is the date of ending FIM, Activity 10 is the date of clinically discharge ready, Activity 11 is the end date. Date 01: onset date; Date 02: acute admission date; Date 03: referral date; Date 04: assessment date; Date 05: clinically rehab ready date; Date 06: episode begin date; Date 07: team planning date; Date 08: date of starting FIM; Date 09: date of ending FIM; Date 10: date of clinically discharge ready; Date 11: end date. Date in red: This demonstrates the date of the activity that is occurring out of a discovered sequence of activities.

The frequency of the nine variants in this case study was 78.46%, 13.08%, 2.31%, 1.54%, 1.54%, 0.77%, 0.77%, 0.77%, and 0.77%, for variants 1 through 9 respectively. The majority of patients (78.46%) fall into Variant 1. Variant 1 comprised 102 cases, where cases followed the same sequence of activities displayed and detailed in Figure 2.

- Of the eight variants (22.54%) representing the rest of the different pathways, variant 2 comprised 17 cases (13.08%), with the case starting FIM (Date 08) before the team planning date (Date 07).
- Variant 3 comprised three cases (2.31%) where acute admission (Date 02) occurred after referral, assessment and being deemed clinically rehabilitation ready (Dates 03, 04, and 05).
- Variant 4 comprised 2 cases (1.54%) where the case was deemed clinically discharge ready (Date 10) before ending FIM (Date 09).
- Variant 5 comprised 2 cases (1.54%) where the onset date (Date 01) was recorded after the acute admission date (Date 01).
- Variant 6 comprised 1 case (0.77%) where clinically discharge ready date (Date 10) was recorded prior to the referral date (Date 02) and subsequent events.
- Variant 7 comprised 1 case (0.77%) where the referral date (Date 03) was after the acute admission date (Date 02).
- Variant 8 comprised 1 case (0.77%) where the end of FIM (Date 09) occurred after the end date for case episode (Date 11).
- Variant 9 comprised 1 case (0.77%) where the episode begin date, team planning date, and date of starting FIM (Dates 6, 7 and 8) occurred before the referral date, assessment date, clinically rehabilitation ready date (Dates 3, 4 and 5) as well as the case starting FIM (Date 08) before the team planning date (Date 07).

In summary, variants 1 and 2 were 91.54% of the total case with the majority of cases (78.46%) in variant 1. Then the remaining variants 3 to 9 were comprised of 7 cases out of 130 (8.46%), with events not conforming to the guidelines process and sequence demonstrated in Figure 2. Summary of pathways demonstrated in Table 5.

Q2: What variation can process mining reveal across stroke rehabilitation patient journeys categorized by patient characteristics (age, gender, and baseline FIM) and across LOS and FIM outcomes?

The current research utilized process mining variant analysis to reveal differences in stroke rehabilitation journeys*.* For illustrative purposes, event log attribute categories for FIM change, LOS and age were developed to illustrate how outputs can be interpreted for stroke rehabilitation. Variant analysis was utilized to discover different pathways patients went through in the stroke rehabilitation journey, and their relationship with age, LOS and FIM change were investigated.

With this analysis, a relationship was found between age, gender, LOS and FIM change, measured by the number of variants. Of those cases, four patients experienced a stroke rehabilitation journey of more than 100 days, warranting further investigation.

**Figure 6.**
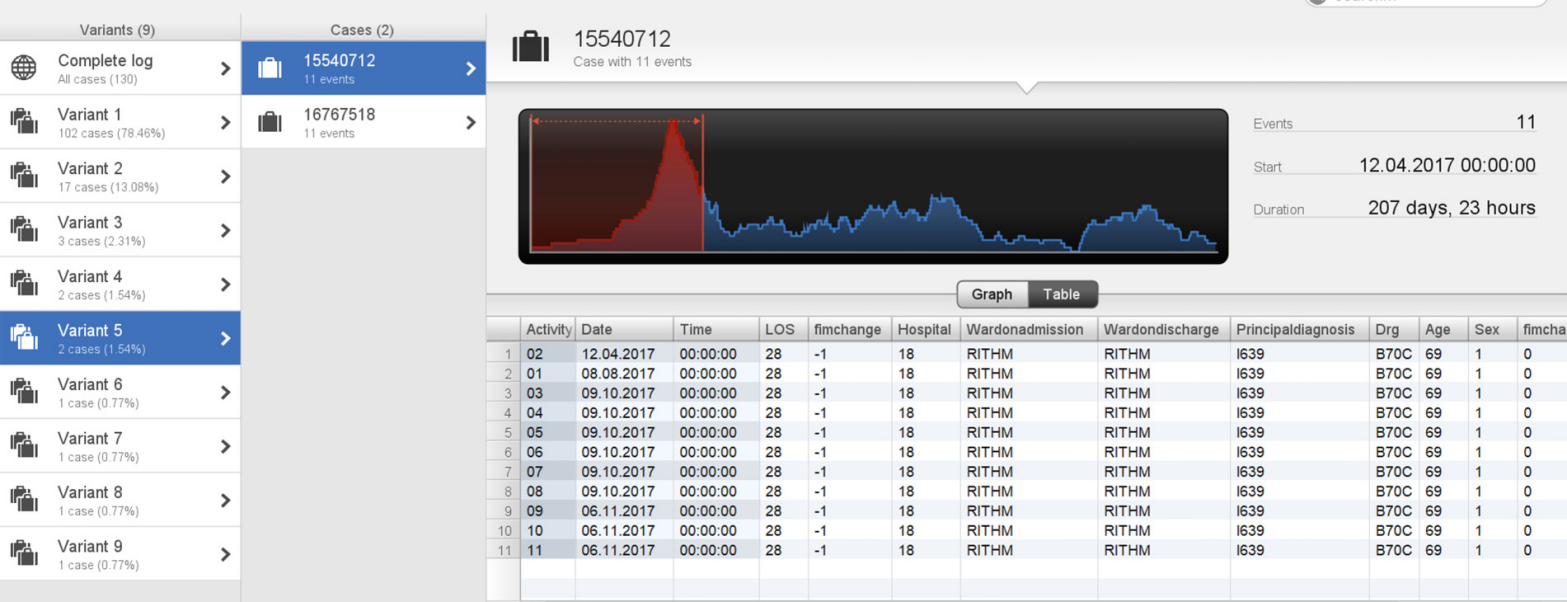
Case 1: patient is in Variant 5 with a total duration of 207 days.

*Note*. Case 1 in Variant 5 has been highlighted in the left column of the figure. Activities 01 to 11 for Case 1 in Variant 5 can be seen in the table in the right bottom columns of the figure. Patients followed a different sequence of activities in their patient journeys, as follows, Activity 02 is the acute admission date, Activity 01 is the onset date, Activity 03 is the referral date, Activity 04 is the assessment date, Activity 05 is the clinically rehab ready date, Activity 06 is the episode begin date, Activity 07 is the team planning date, Activity 08 is the date of starting FIM, Activity 09 is the date of ending FIM, Activity 10 is the date of clinically discharge ready, Activity 11 is the end date. The patient had an acute admission date was 12 April 2017 and then subsequent onset date recorded 8 August 2017, then a referral date of 9 October 2017, then an end date recorded to be 6 November 2017. A long total duration of 207 days and 23 h can be seen in the top right of the figure. The patient recorded a negative FIM change, thus deteriorated after rehabilitation.

Case 1 was in variant 5 and was 69 years of age, with a total duration of 207 days (Figure 6). On further investigation, the case's acute admission date was 12 April 2017 and then the subsequent onset date was recorded as 8 August 2017, then a referral date of 9 October 2017, then an end date recorded to be 6 November 2017. The patient also recorded a negative FIM change, thus deteriorated after rehabilitation. This demonstrates potentially delayed referral followed by a subsequent speedy rehabilitation journey with suboptimal outcomes. Figure 5 demonstrates this patient in variant 5 with a total LOS duration of 207 days.

**Figure 7.**
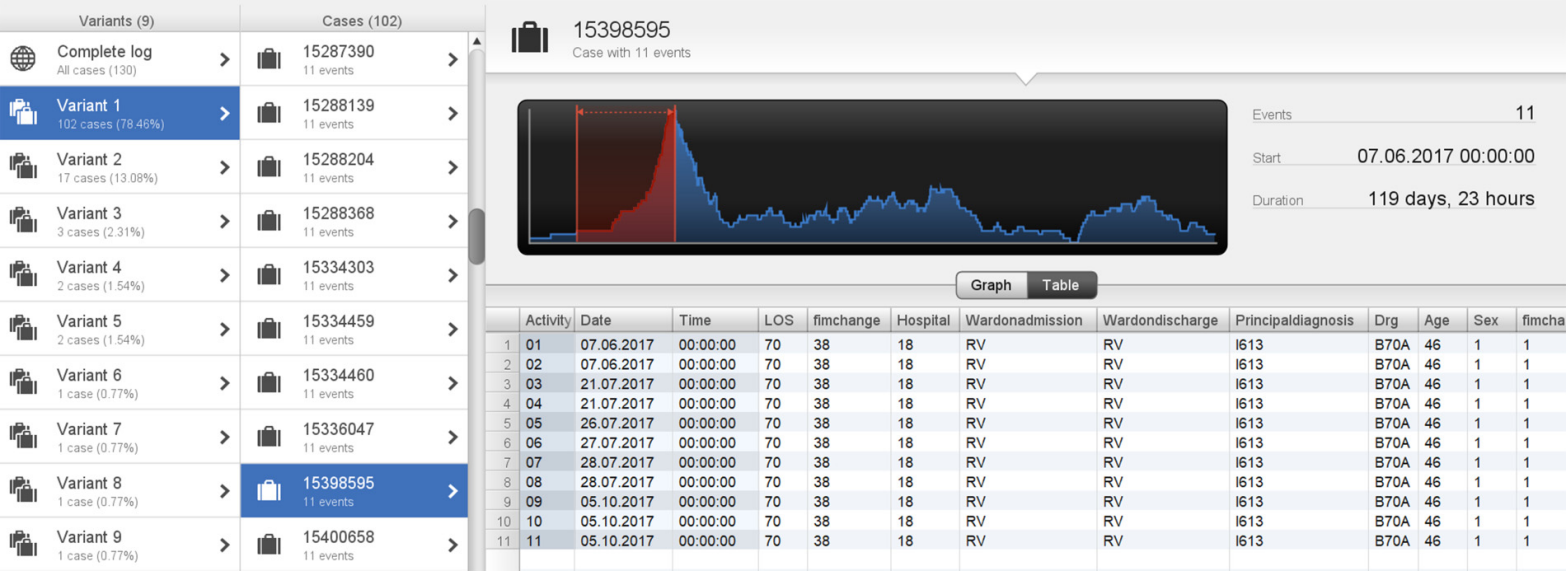
Case 2: patient in variant 1 with a total duration of 119 days.

*Note*. Case 2 in Variant 1 has been highlighted in the left column of the figure. The total duration of 119 days and 23 h can be seen in the top right of the figure. Activities 01 to 11 for Case 2 in Variant 1 can be seen in the table in the right bottom columns of the figure. The patient followed the expected sequence of activities in their patient journeys as follows, Activity 01 is the onset date, Activity 02 is the acute admission date, Activity 03 is the referral date, Activity 04 is the assessment date, Activity 05 is the clinically rehab ready date, Activity 06 is the episode begin date, Activity 07 is the team planning date, Activity 08 is the date of starting FIM, Activity 09 is the date of ending FIM, Activity 10 is the date of clinically discharge ready, Activity 11 is the end date. However, there was a prolonged length of time between StartFIM and EndFIM dates, started FIM on 28 July 2017 and ended on 5 October 2017.

**Figure 8.**
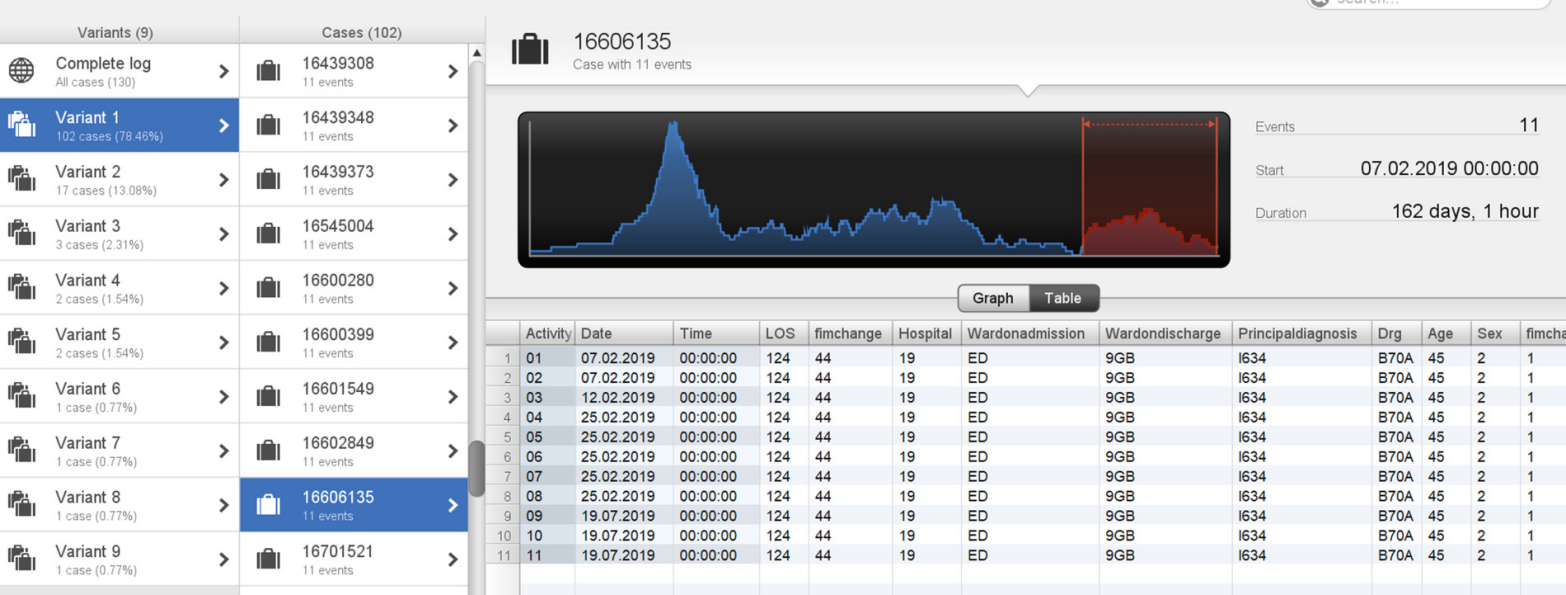
Case 3: patient in Variant 1 with a total duration of 162 days.

*Note*. Case 3 in Variant 1 has been highlighted in the left column of the figure. The total duration of 162 days and 1 h can be seen in the top right of the figure. Activities 01 to 11 for Case 3 in Variant 1 can be seen in the table in the right bottom columns of the figure. The patient followed the expected sequence of activities in their patient journeys as follows, Activity 01 is the onset date, Activity 02 is the acute admission date, Activity 03 is the referral date, Activity 04 is the assessment date, Activity 05 is the clinically rehab ready date, Activity 06 is the episode begin date, Activity 07 is the team planning date, Activity 08 is the date of starting FIM, Activity 09 is the date of ending FIM, Activity 10 is the date of clinically discharge ready, Activity 11 is the end date. However, there was a prolonged length of time between StartFIM and EndFIM dates, 25 February 2019 and ended on 19 July 2019.

**Figure 9.**
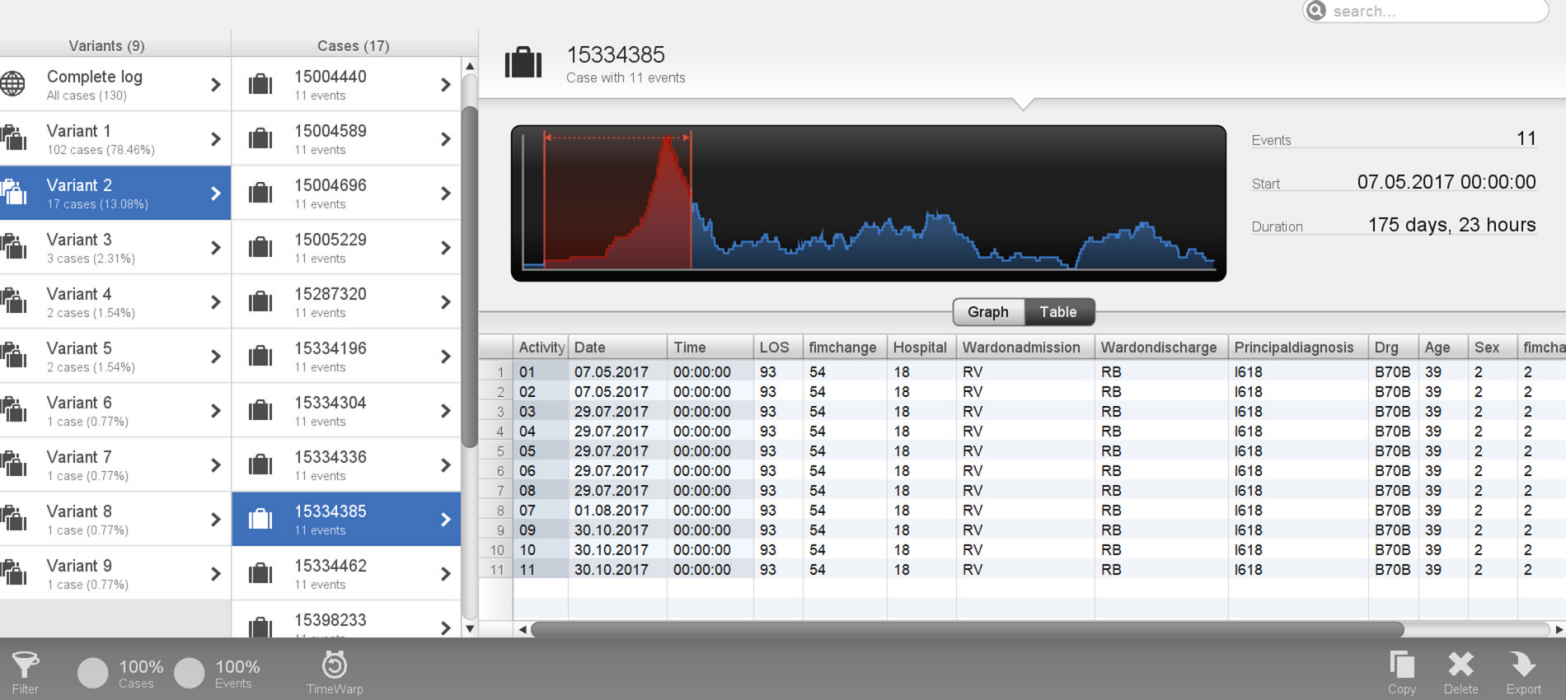
Case 4: patient in Variant 2 with a total duration of 175 days.

*Note*. Case 4 in Variant 2 has been highlighted in the left column of the figure. A total duration of 175 days and 23 h can be seen in the top right of the figure. Activities 01 to 11 for Case 4 in Variant 2 can be seen in the table in the right bottom columns of the figure. Patients followed a different sequence of activities in their patient journeys where the team planning date was after the start FIM date, as follows, Activity 02 is the acute admission date, Activity 01 is the onset date, Activity 03 is the referral date, Activity 04 is the assessment date, Activity 05 is the clinically rehab ready Date, Activity 06 is the episode begin date, Activity 08 is the date of starting FIM, Activity 07 is the team planning date, Activity 09 is the date of ending FIM, Activity 10 is the date of clinically discharge ready, Activity 11 is the end date. However, there was a prolonged length of time between StartFIM and EndFIM dates, the patient started FIM on 29 July 2017 and ended on 30 October 2017.

The other three cases (cases 2–4) were in the age category <50 years aged 39, 45 and 46, with total durations of 119, 162, and 175 days, respectively. Two cases (cases 2 and 3) were in variant 1 and followed the process depicted in Figure 6, however, there was a prolonged length of time between StartFIM and EndFIM dates. Case 2 started FIM on 28 July 2017 and ended on 5 October 2017 and Case 3 started FIM on 25 February 2019 and ended on 19 July 2019 with a total duration of 162 days (Figures 7 and 8). Case 4 was in variant 2, where the team planning date was after the start FIM date. The case started FIM on 29 July 2017 and ended on 30 October 2017. This demonstrates potentially prolonged rehabilitation therapy, based on individual case needs with younger age potentially a contributing factor to additional rehabilitation needs (Figure 9).

**Figure 10.**
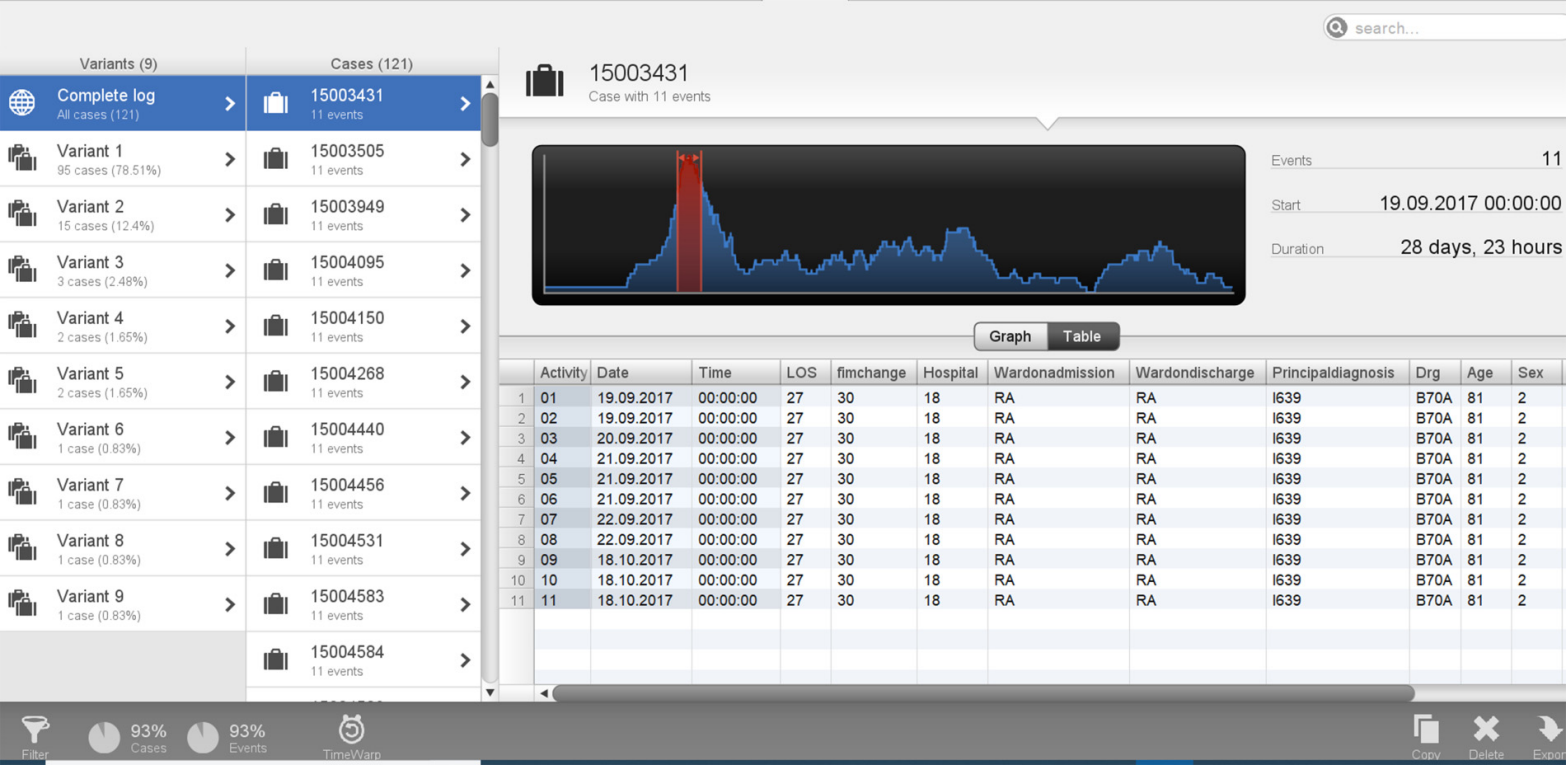
Age category of more than 50 years and process variants.

*Note*. LOS: length of stay. There were nine process variants, of these cases 83% had a LOS < 50 days (LOS Category 1). One hundred and twenty-one (93.1%) patients were more than 50 years. Variants 1 to 9 can be seen in the left column of the figure. Activities 01 to 11 can be seen in the table in the right bottom columns of the figure.

**Figure 11.**
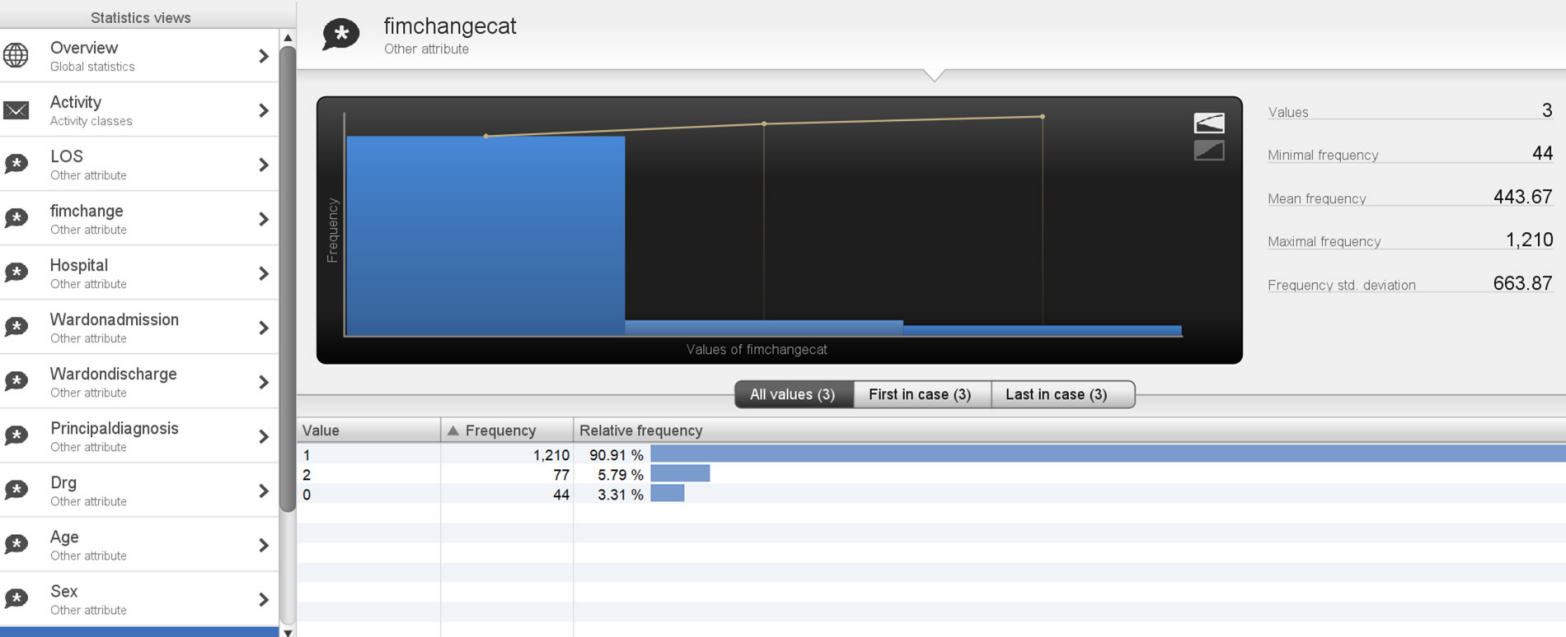
Age category of more than 50 years and outcome measure FIM change.

*Note*. There were nine process variants, and with regard to outcomes, FIM change for the age category of more than 50 years can be seen in the table in the right bottom columns of the figure above. In total, 3.31% of patients had an FIM change between −50 and −1 (Category 0 FIM change), 91.91% of patients had a FIM change between 1 and 50 (Category 1 FIM change), 6% of patients with FIM change > 50 (Category 2 FIM change).

In addition, 93.1% of the total patient episodes were for patients above 50 years of age (age category 2). There were nine process variants, of these cases 83% had a LOS < 50 days (LOS Category 1). In regard to outcomes, 3.31% had a negative FIM change, thus deteriorated (Category 0 FIM change), while 91.91% had an FIM change between 1 and 50 (Category 1 FIM change), and the remaining 5.79% with FIM change > 50 (Category 2 FIM change) (see Figures 10 and 11).

**Figure 12.**
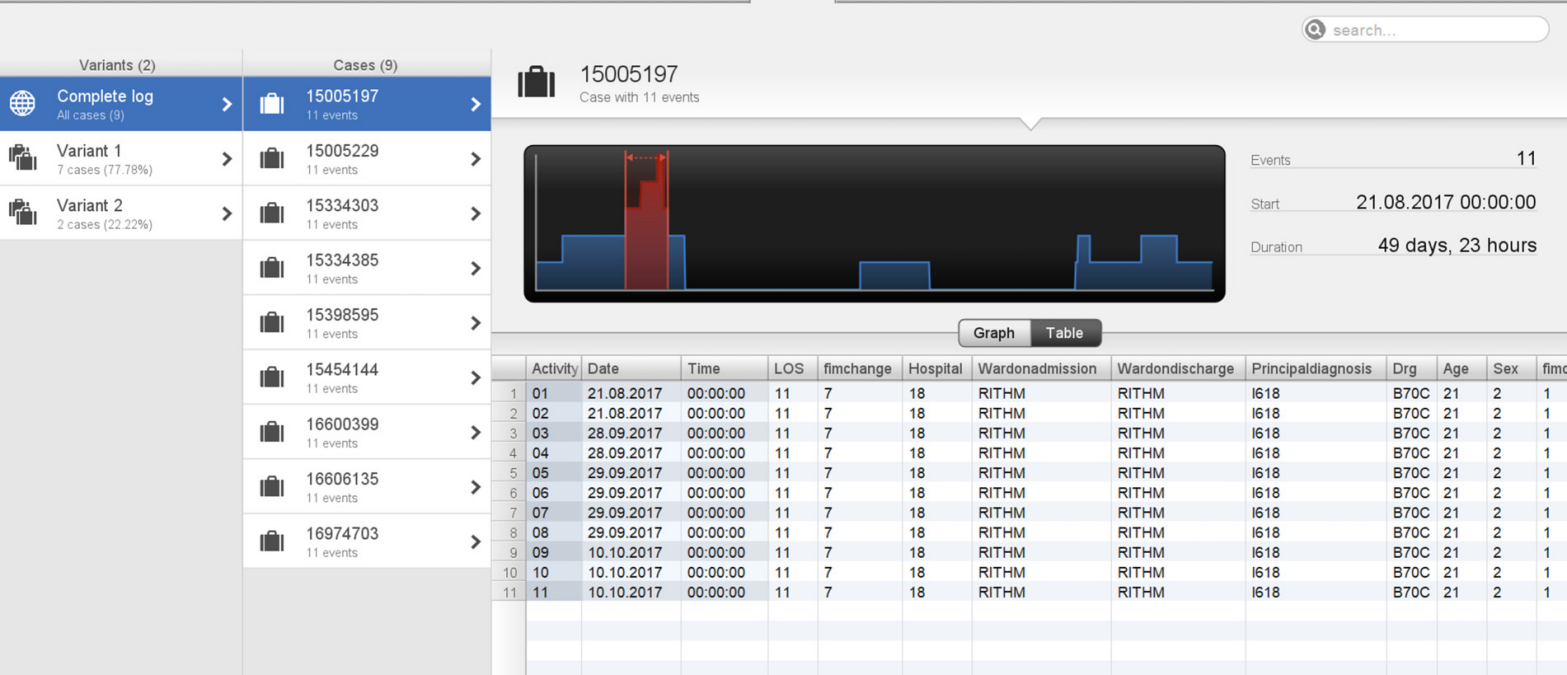
Age category of <50 years and process variants.

*Note*. Patients <50 years old (age category 1) were nine cases, 6.9% of total patient episodes. They demonstrated only two variants, Variants 1 and 2 can be seen in the left column of the figure (6.9%). In total, 77.8% followed the model in Figure 2, while 22.2% of variants followed a different pathway, where team planning was recorded after the FIM start date. Activities 01 to 11 can be seen in the table in the right bottom columns of the figure.

**Figure 13.**
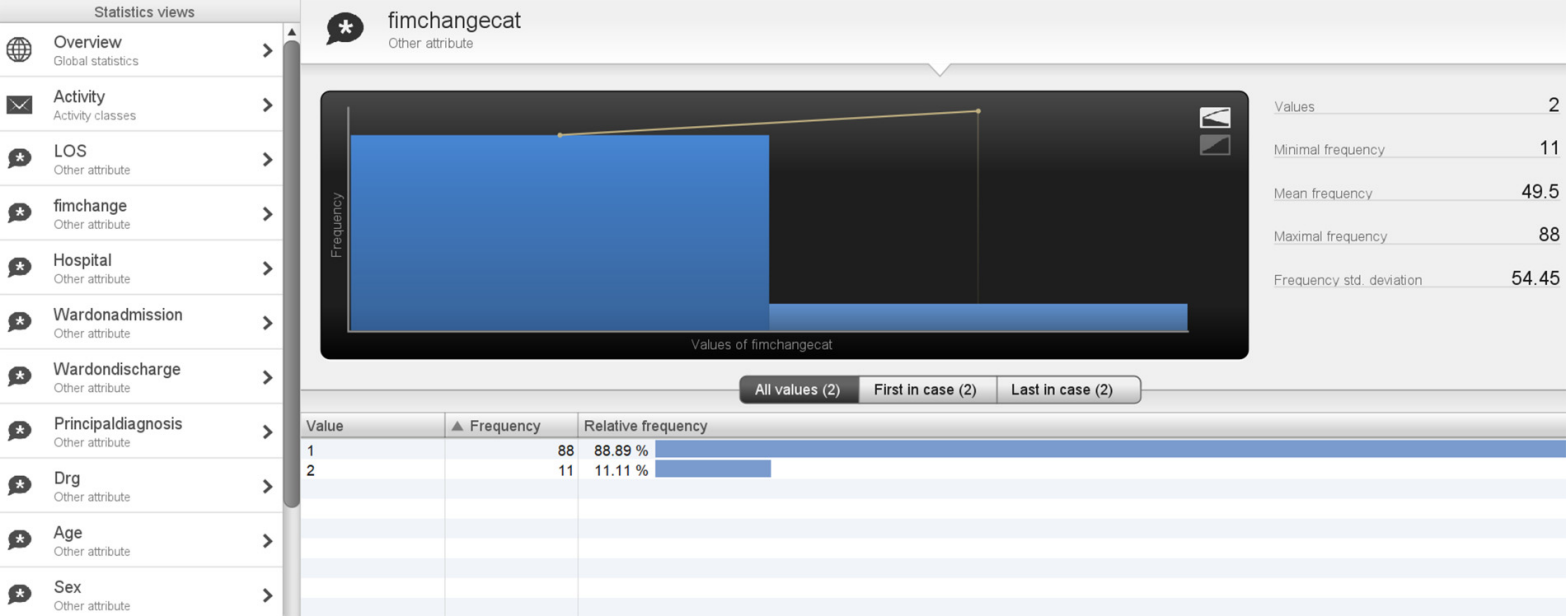
Age category of <50 years and outcome measure FIM change.

*Note*. Patients <50 years old (age category 1) were nine cases, 6.9% of total patient episodes. FIM change for the age category of <50 years can be seen in the table in the right bottom columns of the figure and includes categories 1 and 2 FIM change only. Zero per cent of patients had a FIM change between −50 and −1 (Category 0 FIM change), 88.89% had a FIM change between 1 and 50 (Category 1 FIM change), 11.11% with FIM change > 50 (Category 2 FIM change).

In comparison, patients <50 years old (age category 1) were 6.9% of total patient episodes, and had two process variants, of these cases 77.8% followed the model in Figure 2, while 22.2% variants followed a slightly different pathway, where team planning was recorded after FIM start date. Of these cases, 88.89% had an FIM change between 1 and 50 (Category 1 FIM change), 11.11% had FIM change >50 (Category 2 FIM change) and no patients demonstrated a negative FIM change, that is, none deteriorated (Category 0 FIM change) (see Figures 12 and 13). This highlights that older patients demonstrated lower FIM change improvements, with some deteriorating where 100% of patients deteriorating were >50 years of age.

## Discussion

Process mining is an established methodology for process discovery, conformance, and enhancement.^20^ The main techniques and algorithms that have been utilized in process mining case studies include Heuristics Miner, Fuzzy Miner and Trace Clustering.^35^ CJM is a management technique that demonstrates process paths, and process mining's generated journey maps demonstrate CJs and provide strong analysis of different process paths, variants and touchpoints for consumer end-to-end processes.^4^ Utilizing process mapping and CJ mapping to inpatient stroke rehabilitation data in the current study, the routinely collected clinical data had the ability to demonstrate in great detail the multiple touchpoints the patient went through in their journey. As demonstrated by previous authors,^4,6,18,19^ these journey maps that are generated are a valuable visual tool to analyse inpatient healthcare processes for inpatient stroke rehabilitation patients.

Process mining undertaken in the current research is relatively simple and inexpensive to apply, whereas after extracting and formatting the data to build the event logs as per the process mining tool's (Disco) requirements, the time and cost involved in undergoing process mining through utilizing Disco were not complex and there was a myriad of open-source information available.

In healthcare, various units and systems have different information systems which in many instances work independently, with various event logs across the continuum of a patient journey. Thus in developing CJ maps for patient journeys, information sharing to create a single event log is often challenging.^36^ A significant contribution of this research relates to developing the data linkage between the AROC database and inpatient stroke data, outlining the sequential steps in obtaining, linking, analysing and presenting the process data for stroke rehabilitation patients. This included data extraction and linkage to ensure accurate and complete data to construct the event logs and provide the data structure for storing information to ensure subsequent analysis provides the required results.

In addition, an important aspect of process mining and analyses of healthcare processes is including additional information that can complement the discovered process.^4^ The current study contributed by including and investigating relationships with patient characteristics, including age and gender, as well as patient outcomes, including FIM and LOS. In the current research, the findings demonstrate the methodology of utilizing process mining for process discovery and analysis to identify variants in the sequential journey of stroke rehabilitation patients. Variants in rehabilitation journeys exceeding targeted LOS, FIM changes, and other key outcome measures discovered through process mining warrant further investigations and analysis. Such analysis and results can contribute to the generation of hypotheses for discovering process improvement opportunities for stroke rehabilitation. As an example, in some variants with longer LOS, demonstrating potentially prolonged rehabilitation therapy, analysis and identification of individual case needs related to age, gender, and FIM change could potentially be a contributing factor to additional rehabilitation needs. Another example is variant analysis drilling into individual case levels and analysis of the individual cases and deviations and non-conformance from the described process such as variants two through to nine that would warrant further action in the clinical setting. The addition of more characteristics and outcomes will broaden even more tailored and valuable analysis for patients.^4^

In addition to process analysis, another contribution of the current research through mapping patient journeys and touchpoints is enabling care providers to contribute to the principles of patient-centred care^37^ through better coordination and integration of care across patient journeys as well as more efficient transition and continuity of care. South Australia's eight principles of patient-centred care include respect for the patient's values, preferences and expressed needs, coordination and integration of care, information, communication and education, physical comfort, emotional support, involvement of family, friends and carers, transition, and continuity as well as access to care.^37^ Utilizing process mining approaches leads to identifying and visualizing patient touchpoints across the care continuum, locating non-conformance to guidelines and bottlenecks, as well as subsequent analysis of prolonged patient journeys. Future research generating hypothesis that further investigates and analyses patient-reported experience measures, for example through patient satisfaction surveys,^4^ and outcome measures in touchpoints through stroke rehabilitation journeys can contribute to process improvements in particular touchpoints with suboptimal patient experience and gaps in service quality for better patient-centred care in stroke rehabilitation. In addition, novel algorithms and metrics that could cluster patient journeys, attributes and outcomes, can also provide predictive capabilities for process mining and CJs.^18^

## Limitations

The current research investigated several aspects of the patient journey, including exploring the customer, journey, mapping, goals, touchpoints, and timeline. The current study had some limitations. Firstly, the dataset did not include the patient journey from the emergency department (ED) presentation and throughput through the wards. Nor did the dataset include events or attributes from the outpatient component of the patient's journey (pre-admission and post-discharge), thus limited access to the whole care continuum which is needed to make available a complete patient journey. Secondly, patient experience surveys and scores were not available in the included dataset, thus limiting the assessment into CJ variations and patient satisfaction. Finally, additional aspects of the CJ, including subprocesses, lenses and multimedia were not available as data was generated from deidentified patient records, thus limiting the evaluation of all components of the patient journey. Finally, the process evaluation was only applied within the context of the stroke rehabilitation patient journey.

One challenge in process mining in health is discovering effective methods to gather and measure patient experience and link it with processes to have a complete patient journey map that contributes to better healthcare service quality and patient experience.^4^ There is scope for future research in exploring additional components of the journey map including stages (subprocesses), stakeholder experiences, application of different lenses as well as extraction from different multimedia. Including additional variables to complete the journey map is an area of future research of significance to improve the patient's experience. Finally, future research utilizing several analytical approaches and investigating automation for comparing the results would be beneficial.

## Conclusions and future work

The current research describes a process mining application for stroke rehabilitation patients and demonstrates how to utilize process mining for better understanding and evaluating patient journeys in healthcare, ultimately aiming to improve the quality of care and reduce process variations in stroke rehabilitation journeys. Further research can include expanding the stroke rehabilitation patient journey to explore the complete continuum from ED presentation to discharge and beyond as well as sub-processes and additional attributes along this journey. This may include among others variations in episodes of care, rehabilitation start, ward transfers, allied health services provision, additional outcome measures, additional demographics, discharge destinations, as well as different types of rehabilitation provision (in-patient, day, and at-home). Further research can also include patient satisfaction surveys as well as stakeholder and organizational perspectives to analyse patient touchpoints and interactions with staff. Finally, translating the research into practice through engagement with clinicians would work towards decreasing variations and embedding quality improvement initiatives throughout healthcare organizations.
